# Suggestions to Medical Writers

**Published:** 1900-11

**Authors:** 


SUGGESTIONS TO MEDICAL WRITERS. By GEORGE M. GOULD, M. D. Duodecimo, pp. 185. Philadelphia: The Philadelphia Medical Publishing Co. 1900.

Of all the works which bear the name of Dr. Gould as author, and there are many, none is more valuable, more interesting than this little volume. It is little in size, but big in scope. In no work is Dr. Gould's forceful writing seen to better advantage than in these "Suggestions to Medical Writers," and the most interesting thing about it all is the charming abandon which he exhibits as he plays hob with the vast army of etymologomaniacs—those poor, narrow-visioned beings who rail at the advance in medical orthography, still spell hemorrhage with a diphthong; and claim that "pathological" is "scientific," not wanting to admit what, in their hearts they must know, is correct. Dr. Gould naively asks why it should not be "scientifical." The greatest crime in medical literature today is what Dr. Gould calls a "hodge-podge of Latin and English." There is too much straining for effect; too much sky-rockety exhibition of "scientifical" learning, which when it is all sifted out is in many, many cases neither fish, flesh nor fowl, but a sort of a literary hash of questionable origin. I will confess I am on the mourner's bench today. When I picked up this book for review it was with the idea that it had fallen to my sorry lot to say things about a bit of work which at first glance struck me, to say the least, as being rather bizarre. I started in heroically and found myself deeply interested; and when a reviewer becomes interested he is much like a dramatic critic who is amused; he is no longer a critic, he is an adherent, an endorser, a friend. And from now on I am an humble follower in the footsteps of Dr. Gould, so far as medical English is concerned. He may be wrong in some of his views on other subjects concerning the profession, and in some he might with great grace appear as a reformer; but in medical English he is the leader and should be followed. It is going to be hard work for a lot of us, but it's going to make us consistent. We may not be gaudy and picturesque to the extent of flying a Latin flag on an English ship sailing under old Greek papers, but we will be honest. The introduction to the "Suggestions" is a brilliant bit of flail-flying and the "pernickety sticklers," as Dr. Gould calls the hardshell objectors to advancement, are held up to ridicule and left among the ruins of a mixed-pickle language, which has been through the etymologic rag shops of many centuries. There is quite as much humor as censure in the work; there is common sense pervading the atmosphere of every page and really a careful study of the book reveals very little which could be honestly criticised. Dr. Gould does not approve of foreign words, but there are some which one cannot very well help using. For instance, I said that he was naive. I could not with dignity and without sacrificing the great respect I have for him as a writer, say he was coy or cute, but I could say he was naive and I did—and he is.

The writing of prescriptions in good English is one of Dr. Gould's appeals. Every drug store in the city of Buffalo has several scrap-books, which are truly volumes of shame. They contain the hog-Latin prescriptions of practising physicians. Let me echo the refrain in Dr. Gould's book: "Stick to the English," and be honest.

Time was when the physician was a creature of supposedly supernatural attributes and his calling was a mystery. He wrote his prescriptions in ink carried in a horn at his girdle; and what a wonderful creation that prescription was, made up of odd, fantastic characters and symbols known only to himself and the alchemist,—another queer, old fossil who traded on the superstitions of a people steeped in the fog of medieval mysticism. Dr. Gould objects, with good reason, to the use of Latin in prescriptions. What is the use of it anyway? What is accomplished? Is it to keep from our patients the knowledge of the drug they are taking, or is it because we are in a rut, marked out by the sandal-shod feet of past centuries? In these days of public libraries with medical rooms we cannot conceal from a patient, if he desires to learn it, the fact that he is getting calomel when we write "hydrarg. chlor. mite."

How many doctors are there who can tell the origin of the ℥ and ʒ signs which have for years and years been in use? How many can tell what the ℞ means? A few years ago we were told in the medical colleges—some of them—that this sign was a contraction, or rather a profanation of the old zodiacal sign of Jupiter, and that it had come to us as a sort of a hand-me-down from the ancient physician who used to begin his prescription with what might very properly be termed a voodoo appeal to Jupiter, all of which is the veriest rot. While we must perforce in some cases stick to mercury, we ought to remember that Jupiter is a back number in prescription writing, and eliminate from our minds all the sleight-of-hand tricks which were practised centuries ago. The plea is made that a prescription written in Latin is standard the world over. If one is to practise medicine in a foreign country then Latin may be a necessity; but the number of men in this country who could pass the qualifying medical examinations in any foreign country on the face of the globe, would not make a decent sized crowd at an afternoon tea. An English speaking physician who writes a prescription in Dr. Gould's "hodge-podge" must appear much like the savage who imagines himself civilised, when he adds to his breech clout a plug hat and a liver pad picked up on the beach of his mid-ocean island.

Let the doctor of today read the chapters on Medical Paleography and Psychology of Words. He need not be too radical, but he can be sensible and he will be a writer of better English.

